# Circulating Cell-Free DNA Integrity for Breast and Prostate Cancer: What Is the Landscape for Clinical Management of the Most Common Cancers in Women and Men?

**DOI:** 10.3390/ijms26030900

**Published:** 2025-01-22

**Authors:** Navid Sobhani, Domenico Tierno, Nicola Pavan, Daniele Generali, Gabriele Grassi, Fabrizio Zanconati, Bruna Scaggiante

**Affiliations:** 1 Department of Cancer Biology, The University of Texas MD Anderson Cancer Center, Houston, TX 77054, USA; navid.sobhani@cantab.net; 2 Department of Medicine, Surgery and Health Sciences, University Hospital of Cattinara, University of Trieste, 34149 Trieste, Italy; domenico.tierno@units.it (D.T.); dgenerali@units.it (D.G.); ggrassi@units.it (G.G.); f.zanconati@fmc.units.it (F.Z.); 3 Department of Precision Medicine in Medical, Surgical and Critical Care, University of Palermo, 90127 Palermo, Italy; nicola.pavan@unipa.it; 4 Multidisciplinary Unit of Breast Pathology and Translational Research, Cremona Hospital, 26100 Cremona, Italy; 5 Department of Life Sciences, University of Trieste, 34127 Trieste, Italy

**Keywords:** ALU sequences, breast cancer, circulating cell-free DNA integrity, cfDI, LINE1 sequences, liquid biopsy, prostate cancer

## Abstract

Breast cancer (BC) and prostate cancer (PCa) are major health problems for women and men worldwide. Although therapeutic approaches have increased, the complexity associated with their heterogeneity and progression requires better ways to monitor them over time. Cell-free DNA integrity (cfDI) represents a viable alternative to needle biopsy and has the potential to be representative of cancer at all stages. In addition to the advantages of liquid biopsy in terms of cost and reduced invasiveness, cfDI can be used to detect repetitive DNA elements (e.g., ALU and LINE1), which could circumvent the problem of mutational heterogeneity in BC and PCa. In this review, we summarise the latest findings on cfDI studies in BC and PCa. The results show that cfDI has the potential to improve early detection, metastasis, and recurrence of BC, while limited studies prevent its clinical value in PCa from being fully defined. However, it is expected that further studies in the near future will help to introduce the use of cfDI as another biomarker for the clinical monitoring of BC and PCa patients.

## 1. Introduction

Breast cancer (BC) is a complex and heterogeneous disease [1], which is one of the most common cancers in women in terms of both incidence and mortality [2]. According to Globocan 2024, 2,308,897 new cases and 665,684 deaths are expected worldwide in 2022 [2]. However, thanks to increasing therapeutic options, the prognosis of BC patients has improved considerably in recent years [3]. Tumour grade, hormone receptor status, and human epidermal growth factor receptor 2 (HER2) are the most important cancer-related factors that influence metastatic potential, response to treatment, and prognosis [3]. Metastatic BC (MBC) remains an incurable disease, but early detection can increase survival rates in the early stages [4,5]. Nevertheless, in both cases, a tissue biopsy is urgently required for pathological assessment and for the selection of biomarker-based therapeutic approaches [6]. Unfortunately, standard methods are associated with high costs, invasiveness, and low sensitivity and specificity [7]; therefore, they are not suitable for all patients [8]. Another important limitation is the ability to capture the entire genomic landscape of the tumour, including intratumoural heterogeneity [8,9].

Prostate cancer (PCa) is a widespread malignant disease in men. It is the second most commonly diagnosed cancer and one of the main causes of cancer-related deaths worldwide. In 2022, 1,466,680 new cases and 396,792 deaths were reported worldwide [2]. The incidence of PCa increases with age, with over 85% of diagnoses occurring in men over the age of 60 [10]. In general, for both BC and PCa patients, conventional diagnostic methods, such as tissue biopsies, are invasive and may not fully capture the heterogeneity of the tumour.

Peripheral blood is one of the most important sources of various circulating cell-free nucleic acids, circulating tumour cells (CTCs), and exosomes. One type of nucleic acid in the blood is cell-free DNA (cfDNA), which is derived from DNA fragments released into the peripheral circulation from either dying tumours or normal cells. A special population of cfDNA is the circulating tumour DNA (ctDNA). It is differentially selected on the basis of tumour-specific DNA changes such as gene amplifications, mutations, methylations, and rearrangements [11]. Liquid biopsy, a minimally invasive technique that analyses tumour-derived material such as cfDNA, CTCs, and extracellular vesicles (EVs) in body fluids, has emerged as a promising diagnostic, prognostic, and predictive tool [12,13]. The use of liquid biopsy has completely changed some clinical practices and improved patient outcomes [14]. Circulating tumour DNA (ctDNA), i.e., the part of cfDNA that enters the bloodstream as a result of tumour cell death [15], has been shown to be an informative circulating tool in both BC and PCa [16,17]. For example, early-stage ctDNA clearance has been associated with higher rates of complete pathological response after neoadjuvant treatment and with a low rate of disease relapse in BC [17]. Through longitudinal sampling, ctDNA can monitor response to treatment and individualise optimal treatment in MBC [18]. In localised PCa, liquid biopsy can differentiate between low- and high-grade cancer, helping to decide whether to perform or postpone a tissue biopsy. In advanced stages of disease, it provides prognostic information beyond standard tests such as PSA (prostate-specific antigen) levels and can monitor response to treatment and tumour progression. ctDNA has been used to screen for androgen receptor gene mutations in PCa patients who develop castration-resistant metastatic cancer to achieve prognostic and therapeutic goals or to determine genomic mutational burden in relation to disease aggressiveness and progression [19]. In patients with metastatic castration-resistant disease, specific genomic alterations in ctDNA have been associated with clinical outcomes such as progression-free survival (PFS) and overall survival (OS) [20].

**Figure 1:**
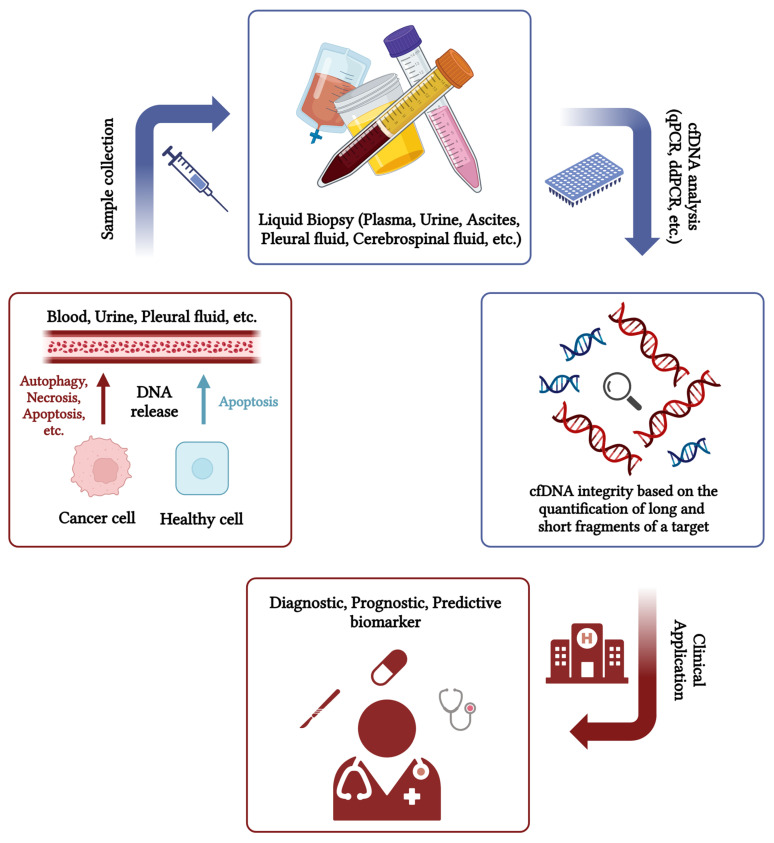
Liquid biopsy for circulating cell-free DNA integrity (cfDI) and its potential clinical applications graphical summary. Created in BioRender. Sobhani, N. (2025) https://BioRender.com/q17z431.

These biomarkers can give information that is either qualitative (e.g., mutation type) or qualitative (i.e., copy numbers of mutations). However, there is a limitation to this method, as not all patients have the same mutations. For example, p53 is mutated in only 26–88% of BC patients. The variation depends on the BC subtype [21]. The same is true for metastatic PCa patients, where the mutation burden involving androgen receptors is 10–20% of metastatic castrate-resistant PCa patients; another tumour-related gene, such as PI3KCA, accounts for 6% of metastatic patients, or germline/somatic mutation of DNA damage repair genes that are found in 15–30% of metastatic patients, half of which is of germline origin [19]. In BC, PIK3CA mutations vary between 25 and 40%, with the highest mutation rate in HR+/HER2− metastasised BC subtypes [22]. In contrast, cfDNA integrity analysis (cfDI) is a potential tool in liquid biopsies that can overcome the limitations of heterogeneity and differential mutation rate in cancer development and progression. The main principle behind cell-free DNA integrity (cfDI) is the fact that normal cells undergo apoptosis, releasing shorter fragments of about 200 bp, whereas tumour cells undergo different death processes, including autophagy or simply necrosis, and release different fragment sizes, often longer than those of the apoptotic process [23,24]. cfDI can be detected by the longer/shorter DNA fragments (Figure 1). Furthermore, the analysis of cfDI has more targets in the samples and is independent of genetic and epigenetic ctDNA. In this review, we provide an overview of the current applications of cfDI tests in BC and PCa and their potential development in clinical practice. We have focused on these two cancers because they are the most common hormone-dependent cancers in women and men, respectively, for which a cost-effective liquid biomarker representative of tumour heterogeneity is a need in many Western countries to aid diagnosis, prognosis, and prediction of response to therapy in the context of precision medicine. To ensure a complete search in PubMed for DNA integrity, we used the following keywords: “circulating” or “cell-free” “DNA” “integrity” “breast cancer” or “prostate cancer”. From the search results, we selected all original articles without time limitations.

## 2. Circulating Cell-Free DNA Integrity (cfDI)

Notwithstanding the great progress that has been made in recent years in the field of BC and PCa, there is still a need for better diagnostic and prognostic tools that can predict the onset, recurrence, or progression of these diseases at an early stage. Liquid biopsy has been proposed as a less invasive and equally valid method compared to needle biopsy. One of the targets in liquid biopsy is known as circulating cell-free DNA integrity (cfDI), which is the fragmentation pattern of cfDNA. Cancer cells can undergo many different types of cell death, including apoptosis, but also necrosis and other types of cell death. Differences in the amount of longer and shorter fragments can be associated with cancer. DNA aberrations in cancer cells also contribute to the difference between longer and shorter fragments that can enter the bloodstream. cfDI was mainly characterised by qPCR using a delta–delta formula based on a threshold cycle (Cp), e(−ΔΔCp × ln(2)), or based on a ratio between the amount of longer fragments and shorter fragments considered representative of the total amount of cfDNA associated with the process of cell death. In fact, DNA fragmentation profiles are different in cancer patients from healthy subjects [25].

### 2.1. cfDI as a Biomarker in Breast Cancer

The following section summarises the cfDI discoveries published in the literature in chronological order (Table 1). In the table, the average age of the cohorts was rounded to decimal numbers. Main results refer to significant results; if no significant results are available, a trend is indicated as non-significant. Clinical value refers to the potential clinical application if significant results are demonstrated. For most studies, qPCR determination of target molecules for cfDI was used, and only methods deviating from qPCR are mentioned in the text.

The first pioneering work was that of Umetani et al. [26] in 2006, who investigated the prognostic ability of cfDI in serum to predict the progression of BC. For this purpose, the authors analysed ALU 260/111 in 83 BC and 51 healthy control women (HC). The results showed that cfDI levels were significantly higher in AJCC stage II, III, and IV BC than in healthy women. They also demonstrated a linear correlation between tumour stage and size, with the ROC curve allowing differentiation between stage II to IV BC and HC (AUC = 0.79). Their data show that cfDI can predict preoperative lymph node metastases [26]. Deligezer et al. then used ALU 247/115 to investigate whether this target could mark the effects of chemotherapy in BC patients. They found that this cfDI did not differ in patients before and after chemotherapy but was significantly higher in BC than in HC [27,28].

In 2012, Agostini et al. [23] analysed preoperative plasma cfDI as a biomarker for regional lymph node metastases in BC. For this purpose, the authors analysed ALU 247/115 in a small sample of BC patients and healthy women. The results showed that cfDI levels were higher in BC patients than in healthy controls. In addition, cfDI showed a moderate ability to detect patients with lymph node metastases [23]. However, Lehner et al. demonstrated that cfDI of ALU 247/115 was not related to the response to neoadjuvant treatment in BC patients [29], but in 2014 Stötzer et al. [24] investigated cfDI as a diagnostic biomarker for BC progression in a large sample population of BC patients. To this end, the authors investigated ALU 247/115 in localised BC, metastatic breast cancer (MBC), benign breast disease (BBD), and healthy female controls (HC). They used two different cfDI determinations: cfDI 1 as the result of the ratio of the qPCR amount of fragments and cfDI 2 as the ratio of qPCR Cp as the ΔΔCp formula. The results showed that cfDI 1 was significantly higher in HC than in BBD and higher in BC and MBC than in BBD. The cfDI 2 confirmed higher values in HC than in BBD and was higher in MBC than in local BC and BBD. However, both cfDI values were not suitable to identify BC compared to HC. On the contrary, it should be noted that the concentrations of ALU fragments allowed a better differentiation between BC and HC (AUC > 95%) and that for MBC, the classical CA 15-3 and CEA had the best diagnostic value [24]. Interestingly, during the same year, Madhavan et al. [30] evaluated cfDI as an early diagnostic biomarker in a large cohort of primary BC (PBC) and MBC patients, using other targets. The authors studied ALU 260/111 and LINE1 266/97 in a large cohort of MBC, PBC, and healthy females (HC). In contrast to the other two groups, the results obtained from this group showed that cfDI was significantly lower in PBC patients than healthy controls. The ROC curves showed a discriminating ability to distinguish HC from PBC (AUC = 0.75), HC from CTC-positive MBC (AUC = 0.93), and HC from CTC-negative MBC (AUC = 0.81). Also, discrimination ability was found for PBC from CTC-negative and CTC-positive MBC (AUC = 0.71 and 0.86, respectively) and for CTC-negative MBC from CTC-positive MBC (AUC = 0.93). In MBC the lowest level was shown in CTC-positive MBC, and the cfDI correlated with worse PFS and OS too [30].

Iqbal et al. [31] evaluated cfDI as a prognostic biomarker for BC prediction. For this purpose, the authors investigated ALU 247/115 in PBC and healthy females. The results contemplated that cfDI was significantly higher in PBC patients than HC. Interestingly, tumour size and cfDI together showed a marked association with disease-free survival (DFS) [31].

Kamel et al. [32] investigated the diagnostic potential of cfDI in BC using *β-actin* fragments (400 bp and 100 bp) in a large group of BC patients at different stages prior to therapeutic intervention. They found cfDI *β-actin* 400/100 was significantly higher in BC patients with respect to benign disease patients (BBD) or matched HC. The ROC curves of cfDI showed 95% CI for discriminating BC from BBD or HC. Also, cfDI was shown to increase with the increase in BC stage, although it did not relate with histopathological type or grade [32].

Maltoni et al. [33] investigated the role of cfDI as a prognostic biomarker using different targets such as *BCAS* 1 266/129, *MYC* 264/128, and *PIK3CA* 274/129 in relapsed BC, non-relapsed BC, and HC. The results showed that cfDI of *MYC* and of *PIK3CA* were significantly lower in patients than in HC, but no significant results were obtained in distinguishing between relapsed and non-relapsed BC patients [33].

Wang et al. [34] investigated the cfDI of ALU 260/111 in non-metastatic BC compared to patients with benign breast disease. They found that cfDI was significantly lower in BC patients than in benign controls. In addition, they found that the cfDI of ALU 260/111 had a ROC curve with an AUC of 0.67 and was a better biomarker than CTCs, cfDNA concentration, and CEA153. Interestingly, cfDI and CTCs together improve sensitivity and specificity and reduce the false positivity of cfDI alone from 50% to 10.7% [34]. Cheng et al. [35] showed that cfDI was lower in a large group of recurring BCs compared to a non-recurring BC group. To this end, the authors analysed ALU 260/111 and LINE1 266/97 and showed that the cfDI of ALU, LINE1, or both in the ROC had an AUC of 0.71, 0.704, and 0.732, respectively. These data also suggested that cfDI was an independent marker of recurrence in BC [35]. Cheng et al. [36] then investigated cfDI as a biomarker for response to therapy by measuring ALU 260/111 and LINE1 266/97 in a large group of MBC patients. The results showed an increase in cfDI (*p* = 1.21 × 10^−7^ for ALU and *p* = 1.87 × 10^−3^ for LINE1) after one cycle of therapy in MBC patients. Of note, cfDI was an independent prognostic marker [36]. Then, Tang et al. [37] investigated the function of cfDI as a diagnostic biomarker in BC by ALU 247/115 in small sample groups of BC, BBD, and HC. The results showed that cfDI was significantly higher in BC than in BBD and HC (cfDI did not differ between BBD and HC). The ROC curve showed an AUC of 0.97, and cfDI correlated with lymph node metastasis and tumour stage [37]. Salimi et al. [38] investigated the role of cfDI biomarkers for BC progression in triple-negative breast cancer (TNBC). The authors analysed the ratio of *β-actin* 394 bp/*β-actin* 99 bp in TNBC and non-TNBC patients as well as in healthy women. The results showed that cfDI was significantly higher in TNBC and non-TNBC patients than in healthy controls and was associated with lympho-nodal metastasis and tumour stage. In particular, the ROC curve showed an AUC = 0.997. Finally, the cfDI significantly increases in TNBC compared to non-TNBC, thus discriminating between the two BC types [38].

Wang et al. [39] investigated whether cfDI of ALU 260/111 and LINE1 266/97 can predict response to neoadjuvant chemotherapy (NACT) in a small group of BC patients. The results showed that cfDI was significantly higher in patients after NACT than in patients before NACT. Interestingly, the increase in cfDI in NACT-treated patients was associated with tumour shrinkage and a reduction in Ki67 levels [39]. Miao et al. came to the opposite conclusion by using a cfDI determined by LINE1 259/97 in a study population of young BC patients, most of whom had high TNM and tumour size and were undergoing adjuvant chemotherapy. They found that cfDI was higher in BC patients before chemotherapy than in BBD or HC. Then, they proved that cfDI decreases after chemotherapy, but they collected blood samples 4 weeks after the completion of chemotherapy [40].

Arko-Boham et al. [41] investigated the role of cfDI of ALU247/115 as a biomarker to identify BC and prostate cancer compared to healthy individuals in small populations. The results showed that cfDI levels were lower in BC than in HC but without statistical significance. In contrast, a statistically higher cfDI value was found in stage II BC [41].

Hussein et al. [42] investigated the role of cfDI ALU 247/115 as a diagnostic and prognostic biomarker in a small group of BCs. The results showed that ALU 247/115 was significantly higher in BC patients than in HC with a ROC AUC = 0.825, but it could not be differentiated between metastatic and non-metastatic patients [42]. Park et al. [43] investigated the role of cfDI of ALU 263/58 as a diagnostic biomarker in BC patients and HC. It was found that the ALU-derived cfDI was significantly higher in patients compared to controls with an AUC of 0.724. However, the methylation status of the LINE1 target provided a better test to differentiate cases from healthy controls [43].

Lamminaho et al. [44] analysed cfDI in a large cohort of non-metastatic BC patients at the time of diagnosis. The cfDI was determined by the ratio of cfDNA fragments greater than 1000 bp to those with less than 1000 bp, measured by electrophoretic separation using the ScreenTape D5000 system. The results showed that a higher cfDI was correlated with significantly poorer survival, but only at a follow-up of 10 years. They showed that a high cfDI was an independent prognostic factor for OS and Breast Cancer Specific Survival (BCSS). This was seen in ER + BC patients in the multivariate analysis. Interestingly, a multivariate logistic regression analysis with cfDI, tumour characteristics, and age at diagnosis strongly improved the predictive results [44].

Adusei et al. [45] investigated in a small cohort of BC patients whether cfDI can be used to predict response to chemotherapy using ALU 247/115. The results showed that cfDI increased after the third cycle of chemotherapy (T2) compared to the second cycle (T1), but without statistical significance. No statistical significance was also found in cfDI of BC cases compared to HC [45].

Cirmena et al. [46] used the quantification of electrophoretic fragments of cfDNA from plasma to evaluate cfDI as a biomarker for response to neoadjuvant chemotherapy (NACT). To this end, the authors used the highly sensitive D1000 Screen Tape Station to measure fragments of 321 to 1000 bp versus fragments of 150 to 220 bp in a small sample of BC patients and HC. The results showed that at time 2 after NACT treatment, cfDI was significantly higher in pathological complete responders than in non-complete responders. Of note, cfDI with magnetic resonance imaging data analysed by ROC showed a predictive value for complete response of 0.875 [46].

In an observational study, Elhelaly et al. [47] investigated the role of cfDI ALU 247/115 as a biomarker for the early detection of BC and the discrimination of BBD. In their cohort of age-matched cases and controls, cfDI was significantly higher in BC than in BBD or control, but it did not discriminate between BBD and HC. The ROC showed an AUC of 0.727 [47].

Hafeez et al. [48] investigated cfDI as a prognostic biomarker for BC. To this end, the authors analysed ALU 247/115 in a small population study of BC, BBD, and healthy women. The results showed that cfDI was significantly higher in BC and BBD than in HC. cfDI was higher in early-stage BC and advanced BC than in BBD, but without statistical significance. To distinguish BC from HC, the ROC of cfDI showed an AUC = 0.71 [48]. Then, Nair et al. [49] analysed cfDI of ALU 247/115 as a prognostic factor in 167 BC patients with different molecular subtypes and stages of disease. They found that cfDI was significantly higher in preoperative BC patients than in postoperative patients, and interestingly, higher cfDI was associated with a mild immune infiltrate and thus a poorer prognosis. This evidence was confirmed by the negative correlation with DFS: DFS decreased from 82 months to 58 months in BC patients with high cfDI values. However, the authors emphasised that higher ALU 247 bp levels may be an independent factor for BC prognosis [49]. In contrast to other groups using qPCR, Bortul et al. [50] investigated cfDI with droplet digital PCR (ddPCR) in a large cohort of BC in 2023. For this purpose, they detected ALU 260/111 and LINE-1 266/97 with ddPCR in 106 BC and 103 HC. The results showed that both cfDI were significantly lower in BC compared to HC. In fact, cfDI LINE1 proved to be more accurate in discriminating cases from controls in ROC (AUC = 0.77) [50].

In 2024, Celik et al. [51] analysed for the first time ccfDNA levels, mtDNA, and DNA integrities together in a small sample of BC patients undergoing NACT compared to healthy controls. The authors investigated whether cfDI of ALU 247/ALU115 and mitochondrial nicotinamide dinucleotide adenine dehydrogenase 4 and 1 (ND4/ND1) can be used to predict response to chemotherapy alone or together with other biomarkers. cfDI ALU 247/ALU 115 was not significantly different in patients before treatment compared to controls but was significantly lower in patients after treatment compared to controls. In contrast, the copy number of mtDNA (ND4/ND1) was significantly higher in patients than in controls both before and after treatment [51]. Also, in 2024, Giro et al. [52] investigated cfDI ALU 247/ALU 115 in a small sample of BC patients 15 days after neoadjuvant chemotherapy. They found that cfDI was significantly higher in patients who achieved a pathological complete response (pCR) and correlated significantly with disease-free survival (DFS) [52]. Finally, Gameel et al. [53] investigated the potential of cfDI ALU 247/115 in a small sample population of BC, BBD, and HC as a biomarker for predicting recurrence. Although cfDI was higher in BC patients than in HC, the differences were not statistically significant [53].

### 2.2. cfDI as Biomarker in Prostate Cancer

The efficiency of cfDI as a tumour biomarker was also investigated in prostate cancer (PCa), but the number of articles was lower than in BC (9 and 25, respectively). Unfortunately, the standard screening method using PSA analysis has too high sensitivity and too low specificity, which poses a diagnostic challenge as it may lead to overdiagnosis and treatment of latent cancer [54]. Therefore, efforts are focused on the evaluation of other biomarkers such as cfDI, the studies of which are summarised in chronological order in this section (Table 2). Analogously to the BC section, in the table, the average age of the cohorts was rounded to decimal numbers. Main results refer to significant results; if no significant results are available, a trend is indicated as non-significant. Clinical value refers to the potential clinical application if significant results are demonstrated. All studies used qPCR to quantify the longer and shorter fragments for cfDI determination.

In 2006, Boddy et al. [55] investigated cfDI for the first time as a biomarker to differentiate PCa patients from patients with benign prostatic hyperplasia (BPH). They quantified the plasma levels of 356 bp and 105 bp sequences of the leptin gene (LEP) in PCa and BPH patients. Leptin is a pleiotropic peptide hormone secreted by adipose tissues and has been associated with various cancers related to obesity [63]. The authors expressed the cfDI as ∆Ct between the average Cts of LEP 356 bp over 105 bp fragments. This ratio value showed no significant difference between PCa and BPH groups [55]. In the same year, Hanley et al. [56] compared the plasma cfDI of PCa patients with those of three different control groups: (1) healthy volunteers (HC) under 40 years of age (Ctr1); (2) patients with radical prostatectomy and low PSA six months after surgery (Ctr2); (3) patients with negative prostate biopsy (Ctr3). The authors used primer sets to identify fragments of 1.3, 1.8, and 2.4 kb from four genomic loci by qPCR. Each of the twelve resulting amplifications (3 fragments for four loci) was assigned a score of 0 (qPCR negativity) or 1 (qPCR positivity). The cfDI was expressed as the sum of the individual scores and ranged from 0 to 10 (2 conditions were excluded as they did not amplify within the 95% error range). The results showed that PCa patients had a significantly higher cfDI compared to Ctr1 and Ctr2, but not compared to Ctr3. A cfDI cut-off of 7 identified 89 of 123 PCa patients, corresponding to a sensitivity of 69.9%. In addition, in another group of 30 PCa patients with negative age-adjusted PSA, this cut-off identified 19 out of 30 PC (63.3%) that were not detected by PSA analysis [56]. To our knowledge, this is the first article to report promising results on cfDI as a biomarker for PCa. In 2013, Feng et al. [57] investigated cfDI as a biomarker to differentiate between PCa patients and BPH patients. In this case, the authors quantified the plasma levels of ALU 247 bp and 115 bp by qPCR and expressed cfDI as the ratio ALU 247/115. The results showed a significantly higher ALU 247/115 ratio in PCa patients than in BPH patients. This significant difference also persisted in PCa and BPH patients with a PSA level of more than 4 ng/mL. In addition, cfDI showed a sensitivity of 81.7% and a specificity of 78.8% in differentiating PC from BPH with PSA ≥ 4 ng/mL (AUC = 0.910) [57]. Casadio et al. [58] investigated the diagnostic value of cfDI by analysing urine samples from PCa patients and HC. The authors amplified sequences longer than 250 bp of the oncogenes c-MYC, BCAS1, and HER2 by qPCR and expressed the cfDI as the sum of the three resulting quantifications. The analysis revealed a significantly higher cfDI value in the urine of PCa patients compared to HC. At a cut-off value of 0.04 ng/µL, the cfDI value in urine showed an AUC of 0.80 (sensitivity: 0.79; specificity: 0.84), indicating a good accuracy of this value in differentiating PCa from HC [58]. These results emphasise that urine is a valuable alternative liquid biopsy for PCa screening compared to plasma and serum. Similarly, Salvi et al. [59] investigated the efficiency of cfDI in urine to differentiate PCa from benign diseases of the urogenital tract (BDUT). The design of the cfDI analysis was the same as Casadio et al. [58], with the only difference that the oncogenes considered were c-MYC, AR, and HER2. The analysis showed no significant difference between PCa and BDUT. Furthermore, the ROC curve analysis showed a lower AUC for cfDI in urine than for PSA (0.50 vs. 0.84) [59], suggesting that the latter is the better choice to distinguish PCa from BDUT.

Another comprehensive analysis by Fawzy et al. [60] compared cfDI, expressed as the ALU 247/115 ratio, between PCa, BHP, and HC. The qPCR analysis revealed a significantly higher cfDI value for the PCa group than for the other groups. The PCa group was further subdivided into metastatic PCa (MPC) and non-metastatic PCa (nMPCa). In this case, the cfDI value was slightly but significantly lower in MPC than in nMPCa [60].

The diagnostic value of cfDI in differentiating PCa from BHP and HC was further investigated by Khani et al. [61] in an Iranian cohort of PCa and BHP patients and HC subjects. Here, too, the ALU 247/115 ratio was determined as a cfDI parameter. The analysis revealed a higher cfDI in PCa patients compared to BHP patients and HC, confirming the results of Fawzy et al. Interestingly, there was no difference in cfDI between the MPCa patients and the nMPCa patients [61]. Arko-Boham et al. [41] compared the diagnostic value of cfDI in serum in two different hormone-related cancers: PCa and BC. In PCa, the authors analysed the ALU 247/115 ratio in the serum of PCa patients and HC. The results again showed a significantly higher ratio in PCa patients compared to HC. In addition, the cfDI correlated positively with high tumour stage and stage [41].

The most recent article investigating cfDI in PCa was published in 2020 by Condappa et al. [62], who analysed cfDI in the plasma of PCa and BHP patients. As usual in recent years, cfDI was assessed as an ALU 247/115 ratio. The results showed no difference in cfDI between PCa and BHP patients, regardless of the results of other similar studies [62]. This discrepancy could be explained by the smallest analysed patient pool of the studies discussed so far (11 PCa and 9 BHP).

## 3. Discussion

Liquid biopsy is a valid, non-invasive, or minimally invasive method that uses biological fluids to identify biomarkers of tumour development and growth, clonal formation at the site of metastasis and metastases, prognosis of DSF and OS, and can be helpful in predicting response to therapy.

Despite its potential, liquid biopsy faces challenges, including the standardisation of detection methods and the validation of clinical utility. Ongoing research aims to integrate liquid biopsy into the routine treatment of BC and PCa patients to improve early detection, surveillance, and personalised therapy [16,64]. Various studies have quantified total cfDNA levels with the following target genes in both BC and PCa: β-*globin*, β-*2 Microglobin*, *GAPDH*, *hTERT*, ALU, or LINE1. Higher levels of the measured cfDNA could be used to distinguish benign from malignant BC [21,22,23,24,25]. It is worth noting that changes in cfDNA levels can be altered by other pathological conditions such as inflammation and infection, which may influence the results [65]. In addition, diurnal variations in individual cfDNA levels have been demonstrated in both healthy individuals and those with cancer [66]. In contrast, cfDI, which is a ratio between longer and shorter fragments of a target, is in principle less affected by the variability of cfDNA quantity from sample to sample. Therefore, the comparison between groups could be more reliable. Of course, the choice of target also has an important influence.

Certainly, with respect to ctDNA mutation targets, the cfDI can overcome limitations related to the mutational rate. In addition, the cfDI could provide an incredible advantage also in terms of sensibility by using repetitive elements as targets, such as ALU and LINE1, that represent about 10 and 17% of the genome, respectively [67]. Here, the current literature on the importance of cfDI in BC and PCa is analysed, and patients are grouped based on the studies examining the nature of the repetitive elements or target genes that determine cfDI.

**Figure 2:**
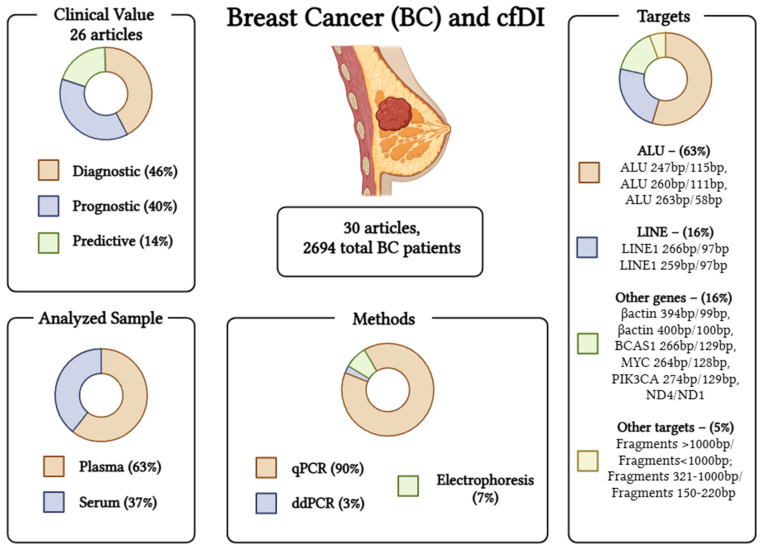
Graphical abstract of the cfDI studies in breast cancer. Created in BioRender. Scaggiante, B. (2025) https://BioRender.com/r57r367.

A graphical summary of the literature results in BC can be found in Figure 2.

In BC, the majority of studies addressed the cfDI of repetitive short interspersed nuclear element (ALU) and long interspersed nuclear element (LINE1) sequences, which accounted for 79% of all studies.

Regarding the cfDI of ALU 260/111 as a diagnostic biomarker, the first study by Umetani showed an increase in serum cfDI in BC at stages from II to IV, accounting for 51 BC patients compared to 51 HC [26]. On the contrary, two other studies on plasma of a large sample population of 188 primary BC and 185 HC showed opposite results with a cfDI decrease in BC compared to HC, confirmed also in 201 MBC compared to HC [30,50]. The discrepancy between the work of Umetani et al. [26] and that of Madhavan et al. [30] and Bortul et al. [50] could be due to differences in the type of samples (serum versus plasma) and in the number and clinicopathological conditions of the cohorts. Consistent with the decrease in cfDI in BC compared to HC, recurrent BC patients had a lower cfDI than non-recurrent BC patients [35]. Interestingly, cfDI ALU 260/111 had prognostic value in terms of tumour stage, metastatic disease, PFS, and OS [26,30,36]. Finally, a higher cfDI was observed in MBC patients after one cycle of chemotherapy [36] and in BC patients with complete response to NACT compared to no-responder patients [39] as a biomarker for the success of the therapy, which measures the increase in circulating DNA due to the death of cancer cells. Differently from ALU 260/111, the cfDI ALU 247/115 showed concordant results in all studies where it was found higher in 522 BC or MBC than in 381 HC or breast benign disease (BBD) [23,31,37,42,48]. A prognostic role of cfDI ALU 247/115 was found to be in accordance with the diagnostic findings for tumour stage and grade, metastasis, and disease-free survival [24,31,37,41,47,49,52,53] and a predictive role for complete response to NACT or chemotherapy [45,51,52]. It is noteworthy that no differences were found in studies using serum or plasma. A study, in which other targets were used in ALU sequences (ALU 263/58), also showed a higher cfDI in BC [39].

LINE1 as a target for cfDI is less studied than ALU, but cfDI LINE1 266/97 showed potential for early detection and screening of BC [50], recurrence [35], and response to therapy [36,39]. Overall, all studies confirmed a lower cfDI in BC at the time of diagnosis compared to HC or at the time of disease recurrence compared to non-recurrence and interestingly showed an increase in cfDI in BC responders to therapy compared to non-responders. The cfDI LINE1 259/97 gave opposite results: The cfDI was higher in BC than in HC or BBD, and consistent with this, it decreased after adjuvant therapy in BC patients who responded to therapy [40]. This raises puzzling questions about these different results when repetitive sequences are used as targets for cfDI. The overall impression is that the target region and the length of the amplified fragment might be the reason for these differences. For example, with more bp in longer fragments, more variability in the results can be attended to because of the susceptibility of circulating DNA to fragmentation and degradation in the pre-analytical phases.

In the studies where specific genes were the target for cfDI at diagnosis, there was also heterogeneity in the results: the cfDI of *β-actin* 400/100 was higher in BC than in HC [32], and that of *β-actin* 394/99 was higher in TNBC than in non-TNBC [38]. However, when *MYC* or *PIK3CA* targets were used, the cfDI was lower in BC than in HC [33].

Finally, emerging evidence of the cfDNA fragmentomic is confirming cfDI as a prognostic [44] and predictive biomarker [46].

**Figure 3:**
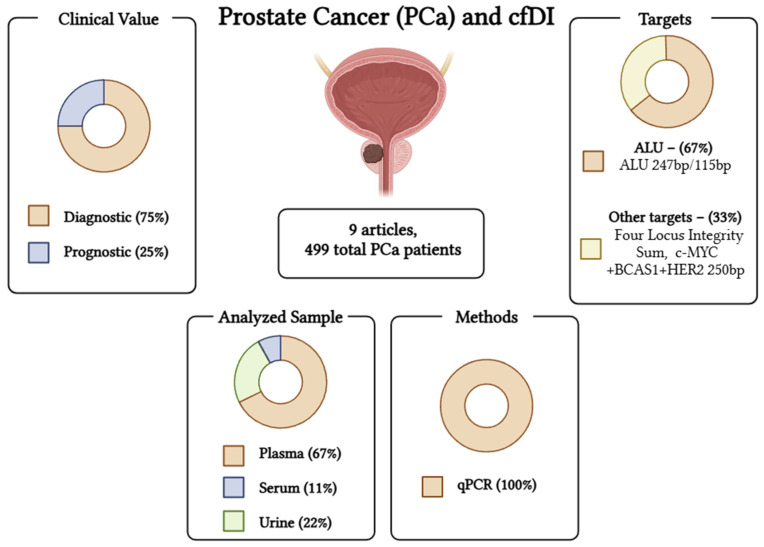
Graphical abstract of the cfDI studies in prostate cancer. Created in BioRender. Scaggiante, B. (2025) https://BioRender.com/t35t139.

A graphical summary of the literature results in PCa can be found in Figure 3.

In the case of PCa, it is initially noticeable that the number of studies on PCa (*n* = 9) is very low compared to BC [30], some of which were not significant (*n* = 3). This is not surprising, as PCa, despite its high incidence in men, is a cancer that affects older people, often as an indolent form, and it is only in the last decade that the interest in early detection of aggressive cancer, active surveillance of localised cancer, and follow-up of patients with castration-resistant forms has increased. Of particular interest are metastatic castration-resistant PCa patients, for whom new targeted therapy options are available to control the disease [68,69]. Of note, most studies in PCa are based on the analysis of ALU repeat sequences. Interestingly, results of cfDI of ALU 247/115 confirm the same trend found in BC: cfDI was higher in PCa than in HC or benign hyperplasia (BPH) [41,57,60], confirmed also by specific gene analysis [58]. In addition, a study using fragmentomic analysis supported a higher cfDI in PCa than in HC [56]. Finally, we would like to point out that in PCa it is possible to analyse cfDI in urine and not in blood, so this test is without any risk [58,59].

## 4. Limitations and Future Directions

The growing therapeutic scenario in the clinical management of BC and PCa patients raises the question of the utility of integrating cfDI analysis into clinical practice. The majority of the studies showed that cfDI differs between patients and controls, emphasising the potential usefulness of this biomarker. Compared to NGS technologies, cfDI has the advantage of being a more sensitive and cost-effective method that, in combination with other biomarkers, could help the clinical management of both BC and PCa patients. Data from the literature have shown that the potential clinical value of cfDI varies depending on the target. Therefore, identifying the best target for BC and PCa is a priority. In addition, the lack of standardised pre-analytical and analytical protocols for cfDI determination remains a limitation. This fact and the nature of the different targets analysed in terms of bp could have an impact on the different results regarding the increase or decrease in cfDI between patients and controls. In addition, for repetitive sequences, which are the most studied targets, there is a lack of robust evidence in large cohorts to deepen the power of cfDI in discriminating BC or PCa patients from HC and benign diseases. Further studies on the role of cfDI as a biomarker for disease progression and response to therapy are also needed.

Overall, the results suggest that cfDI in BC could be used as one of the parameters together with other liquid biopsy biomarkers (e.g., cfDNA, ctDNA, CTCs, etc.) or classical biochemical, radiological, and/or anthropometric data to improve early diagnosis and screening programmes for specific populations (e.g., women with family history) for prognosis and predictive medicine. Since MBC can be cured as a chronic disease, there is also an interest in exploring the prognostic and predictive value of cfDI in MBC. In this regard, cfDI together with ctDNA mutations could be an important complementary tool to obtain relevant information on tumour progression and response to therapy. Very important is the potential of cfDI to differentiate between TNBC and non-TNBC patients, which emphasises the utility of cfDI. It is noteworthy that five studies in BC have found cfDI to be a dynamic biomarker for tracking response to therapy. They show that cfDI levels change during chemotherapy and weeks after the end of chemotherapy [36,39,40,46,52]. An interesting question to address is whether cfDI could be a biomarker for response to immunotherapy and, in particular, to immune checkpoint inhibitors, as tumour mutational burden in ctDNA has been shown to be useful [70]. Unfortunately, to our knowledge, there are no data on the efficacy of cfDI as a biomarker of response to immunotherapy in BC. A future challenge could be to investigate the usefulness of cfDI for predicting and monitoring response to immunotherapy in BC patients. This is justified by the fact that in colorectal cancer, cfDI measured at the β-actin gene (400 bp over 100 bp fragments) predicted response to immunotherapy in terms of PFS, which was higher in a subset of patients with low cfDI than in those with high cfDI levels [71]. Waki et al. [72] also showed that the cfDI of ALU 247/115 was related to vaccine response to vaccination in non-small cell lung cancer. In this case, a high cfDI value was associated with longer survival before and after vaccination.

In contrast, in PCa, there is a paucity of evidence of the potential of cfDI as a diagnostic tool useful for surveillance of high-risk men or for the screening population. However, it should be noted that the majority of the studies analysed examined cfDI ALU 245/115, and the results are consistent with the results of the studies in BC. This encourages further investigations based on BC studies also in PCa patients. Studies on cfDI as a dynamic biomarker in metastatic castration-resistant prostate cancer to monitor the effect of new targeted therapies could be of great interest. Molecular diagnosis prognosis and therapy response by liquid biopsy is an increasing need for the best treatment of BC and PCa patients. Efforts to standardise analysis through precise pre-analytical protocols and the use of high-quality and cost-effective technologies such as ddPCR in conjunction with large multicentre studies are needed to define the potential of cfDI as a biomarker for BC and PCa clinical practice.

## Figures and Tables

**Table 1:** Summary of methods and main results of articles on the integrity of circulating cell-free DNA in breast cancer. ACT: adjuvant chemotherapy; BBD: benign breast disease; BC: breast cancer; cfDI: circulating cell-free DNA integrity; CTC: circulating tumour cell; DFS: disease-free survival; ER+: oestrogen receptor positive breast cancer; HC: healthy control; LN: lymph node; MBC: metastatic breast cancer; NACT: neoadjuvant chemotherapy; no-MBC: non-metastatic BC; OS: overall survival; PFS: progression-free survival; TNBC: triple negative breast cancer.

Number of Patients or Controls	Average/ Interval Age (yr) of Patients or Controls	Analysed Samples	Methods and Targets	Main Results	Clinical Value of cfDI	Citations
83 BC 51 HC	BC = 45 HC = 58	Serum	qPCR on ALU 260 bp, ALU 111 bp. cfDI calculated as the ratio of ALU 260/111.	ALU 260/111 was higher in primary BC patients in stages II, III, and IV than in HC. Moreover, the increase in cfDI was related to the increase in TNM stage of BC.	Diagnostic (BC vs. HC) and Prognostic (Tumour Stage)	Umetani et al., 2006 [26]
41 BC 10 HC	BC = 48 HC = 41	Serum	qPCR on ALU 247 bp, ALU 115 bp. cfDI calculated as ratio of ALU 247/115.	ALU 247/115 did not vary before or after chemotherapy.	No	Deligezer et al., 2008 [27]
73 BC 20 HC	BC = 47 HC = 40	Serum	qPCR on ALU 247 bp, ALU 115 bp. cfDI calculated as ratio of ALU 247/115.	ALU 247/115 was higher in BC (prior to adjuvant chemotherapy) than in HC.	Diagnostic (BC vs. HC)	Deligezer et al., 2008 [28]
39 BC 49 HC	BC = 64 HC = No data	Plasma	qPCR on ALU 247 bp, ALU 115 bp. cfDI calculated as the ratio of ALU 247/115.	ALU 247/115 was higher in primary BC than in HC.	Diagnostic (BC vs. HC)	Agostini et al., 2012 [23]
65 BC	BC = 47	Plasma	qPCR on ALU 247 bp and ALU 115 bp. cfDI calculated as the ratio of ALU 247/115 by two different algorithms (cfDI 1 and cfDI 2).	ALU 247/115 did not predict the response to NACT as either cfDI 1 or cfDI 2.	No	Lehner et al., 2013 [29]
65 BC 47 MBC 12 BBD 28 HC	BC = 47 MBC = 61 BBD = 45 HC = 42	Plasma	qPCR on ALU 247 bp and ALU 115 bp. cfDI calculated as the ratio of ALU 247/115 by two different algorithms (cfDI 1 and cfDI 2).	ALU 247/115 cfDI 1 was lower in BBD than in HC; it was higher in local BC and MBC than in BBD. cfDI 2 was lower in BBD than in HC and lower in local BC than in MBC. It is noteworthy that both cfDI 1 and cfDI 2 did not differentiate between local BC and HC.	Prognostic (Metastatic Disease)	Stötzer et al., 2014 [24]
201 MBC 82 BC 100 HC	MBC = 59 BC = 55 HC = No data	Plasma	qPCR on ALU 260 bp, ALU 111 bp, LINE1 266 bp, and LINE1 97 bp. cfDI calculated as the ratio of ALU 260/111 and LINE1 266/97.	ALU 260/111 and LINE1 266/97 were lower in primary BC, CTC-negative and CTC-positive MBC patients than in HC. In MBC, a lower cfDI was correlated with a worse PFS and OS.	Diagnostic (BC or MBC vs. HC) and Prognostic (Metastatic Disease and Grade; PFS and OS)	Madhavan et al., 2014 [30]
148 BC 51 HC	BC = 45 HC = 58	Serum	qPCR on ALU 247 bp and ALU 115 bp. cfDI calculated as the ratio of ALU 247/115.	ALU 247/115 was higher in primary BC patients compared to HC. Of note, cfDI decreased after surgery and was related to DFS.	Diagnostic (BC vs. HC) and Prognostic (DFS)	Iqbal et al., 2015 [31]
95 BC 95 BBD 70 HC	BC = 58 BBD = 55 HC = 56	Plasma	qPCR on *β-actin* 400 bp and 100 bp fragments and calculated as the ratio of *β-actin* 400/100.	*β-actin* 400/100 was higher in BC than in BBD or HC.	Diagnostic (BC vs. BBD or HC)	Kamel et al., 2016 [32]
58 relapsed BC 21 non-relapsed BC 10 HC	Relapsed BC = 57 non-relapsed BC = 59 HC = No data	Serum	qPCR on *BCAS1* 266 bp and *BCAS1* 129 bp, *MYC* 264 bp and *MYC* 128 bp, and *PIK3CA* 274 and *PIK3CA* 129 bp. cfDI calculated as the ratio of *BCAS1* 266/129, *MYC* 264/128, and *PIK3CA* 274/129.	*MYC* 264/128 and *PIK3CA* 274/129 were lower in BC patients than in HC.	Diagnostic (BC vs. HC) (only for cfDI *MYC* and *PIK3CA*)	Maltoni et al., 2017 [33]
84 no-MBC (early BC = 57; locally advanced BC = 27) 30 BBD	No-MBC = 49 BBD = 42	Plasma	qPCR on ALU 260 bp, ALU 111 bp. cfDI calculated as the ratio of ALU 260/111.	ALU 260/111 was lower in no-MBC than BBD; cfDI plus CTCs can better differentiate no-MBC from BBD.	Diagnostic (no-MBC vs. BBD)	Wang et al., 2017 [34]
175 Non-recurrent BC 37 Recurrent BC	Non-recurrent BC = 55 Recurrent BC = 57	Plasma	qPCR on ALU 260 bp, ALU 111 bp, LINE1 266 bp, and LINE1 97 bp. cfDI calculated as the ratio of ALU 260/111 and LINE1 266/97.	ALU 260/111 and LINE1 266/97 were lower in recurrent BCs than in non-recurrent BCs.	Prognostic (Recurrent BC vs. non-recurrent BC)	Cheng et al., 2017 [35]
268 MBC	MBC = No data	Plasma	qPCR on ALU 260 bp, ALU 111 bp, LINE1 266 bp, and LINE1 97 bp. cfDI calculated as the ratio of ALU 260/111 and LINE1 266/97.	ALU 260/111 and LINE1 266/97 were reduced in MBC with visceral metastasis and increased in MBC after one cycle of endocrine therapy.	Prognostic (Metastatic Disease) and Predictive (Response to Therapy)	Cheng et al., 2018 [36]
40 BC 40 BBD 40 HC	BC = 48 BBD = 46 HC = 45	Serum	qPCR on ALU 247 bp and ALU 115 bp. cfDI calculated as the ratio of ALU 247/115.	ALU 247/115 was higher in BC than in BBD and HC and correlated with LN metastasis and tumour stage.	Diagnostic (BC vs. BBD or HC) Prognostic (including LN Metastasis and Tumour Stage)	Tang et al., 2018 [37]
46 TNBC 50 non-TNBC 50 HC	TNBC = 47 non-TNBC = 41 HC = 48	Plasma	qPCR on *β-actin* 394 bp and *β-actin* 99 bp. cfDI calculated as the ratio of *β-actin* 394/99.	*β-actin* 394/99 was higher in TNBC and in non-TNBC than in HC and correlated with the LN and TN stage.	Diagnostic (TNBC and non-TNBC vs. HC) and Prognostic (LN Metastasis and Tumour Stage)	Salimi et al., 2019 [38]
29 BC	BC = 44	Plasma	qPCR on ALU 260 bp, ALU 111 bp, LINE1 266 bp, and LINE1 97 bp. cfDI calculated as the ratio of ALU 260/111 and LINE1 266/97.	ALU 260/111 and LINE1 266/97 were higher in BC patients after NACT than before NACT.	Predictive (Response NACT)	Wang et al., 2019 [39]
110 BC 35 BBD 90 HC	BC = 35 BBD = 34 HC = 32	Plasma	qPCR on LINE1 259 bp, LINE1 97 bp. cfDI calculated as the ratio of LINE1 259/97.	LINE1 259/97 was higher in BC than BBD and HC; LINE1 259/97 was lower in BC after ACT than before ACT.	Diagnostic (BC vs. BBD or HC) Predictive (before and after ACT)	Miao et al., 2019 [40]
32 BC 32 HC	BC = 51 HC = 53	Serum	qPCR on ALU 247 bp and ALU 115 bp. cfDI calculated as the ratio of ALU 247/115.	ALU 247/115 was significantly higher in BC stage III than in BC stage II.	Prognostic (Tumour Stage)	Arko-Boham et al., 2019 [41]
40 BC 10 HC	BC = 51 HC = 62	Plasma	qPCR on ALU 247 bp and ALU 115 bp. cfDI calculated as the ratio of ALU 247/115.	ALU 247/115 was higher in BC than in HC.	Diagnostic (BC vs. HC)	Hussein et al., 2019 [42]
64 BC 49 HC	BC = 49 HC = 64	Plasma	qPCR on ALU 263 bp and ALU 58 bp. cfDI calculated as the ratio of ALU 263/58.	ALU 263/58 was higher in BC patients than HC.	Diagnostic (BC vs. HC)	Park et al., 2021 [43]
204 non-MBC	BC = 55	Serum	Electrophoretic analysis with the ScreenTape D5000 System to detect fragments > 1000 bp and <1000 bp. cfDI calculated as the ratio of peak areas of fragments > 1000 bp/<1000 bp.	Higher cfDI correlated with poorer survival at 10-year follow-up in ER + BC.	Prognostic (OS in ER+)	Lamminaho et al., 2021 [44]
32 BC 32 HC	BC = 51 HC = 62	Serum	qPCR on ALU 247 bp and ALU 115 bp. cfDI calculated as the ratio of ALU 247/115.	ALU 247/115 increased after the third cycle of chemotherapy but was not significant. No significant difference in cfDI before and after chemotherapy.	No	Adusei et al., 2021 [45]
38 BC 6 HC	BC = 49 HC = No data	Plasma	High Sensitivity D1000 ScreenTape Station on fragments of 321 to 1000 bp and 150 to 220 bp. cfDI calculated as the ratio of fragments of 321 to 1000 bp fragments/150 to 220 bp fragments.	At t2, the cfDI increased and correlated with the achievement of a pathological complete response after NACT. The combination of cfDI and MRI increases the predictive value.	Predictive (Response to NACT)	Cirmena et al., 2022 [46]
50 BC 50 BBD 50 HC	BC = 49 BBD = 48 HC = 46	Serum	qPCR on ALU 247 bp and ALU 115 bp. cfDI calculated as the ratio ALU 247/115.	ALU 247/115 was significantly higher in BC than BBD and HC and correlated with LN metastasis and tumour stage.	Diagnostic (BC vs. BBD or HC) and Prognostic (LN Metastasis and Tumour Stage)	Elhelaly et al., 2022 [47]
20 BC 20 BBD 60 HC	BC = 25–70 BBD = 25–70 HC = 20	Serum	qPCR on ALU 247 and ALU 115. cfDI calculated as the ratio of ALU 247/115.	ALU 247/115 was higher in BC than in HC. It also distinguishes between HC and BBD.	Diagnostic (BC vs. HC and BBD vs. HC)	Hafeez et al., 2023 [48]
167 BC (55% HR+, 26% TNBC) (8% MBC)	BC = 56	Plasma	qPCR on ALU 247 bp and ALU 115 bp. cfDI calculated as the ratio of ALU 247/115.	ALU 247/115 was lower in the post-operative BC than in pre-operative BC; ALU 247/115 was higher in MBC than post-operative BC; ALU 247/115 was higher in HER2+ than TNBC; ALU 247/115 was higher in mild immune-infiltrated ER− than dense immune-infiltrated ER−; high ALU 247/115 correlated with low DFS.	Prognostic (post-operative BC vs. MBC; HER2+ vs. TNBC; mild immune infiltrated ER− vs. dense immune infiltrated ER−; DSF)	Nair et al., 2023 [49]
106 BC 103 HC	BC = 62 HC = 52	Plasma	ddPCR on ALU 260 bp, ALU 111 bp, LINE1 266 bp, and LINE1 97 bp. cfDI calculated as the ratio of ALU 260/111 and LINE1 266/97.	ALU 260/111 and LINE1 266/97 were lower in BC compared to HC. LINE1 266/97 was the most useful biomarker.	Diagnostic (BC vs. HC)	Bortul et al., 2023 [50]
36 BC 21 HC	BC = 50 HC ≤ 50	Plasma	qPCR on ALU 247 bp, ALU 115 bp, mtND4, and ND1. cfDI calculated as the ratio of ALU 247/115 and mt ND4 and ND1 as 2-DCt.	In the post-treated patients, ALU 247/115 and mtDNA (ND4/ND1) decreased compared to the pre-treated patients, but they were not significant.	No	Cèlik et al., 2024 [51]
38 BC	BC = 46	Plasma	qPCR on ALU 247 bp and ALU 115 bp. cfDI calculated as the ratio of ALU 247/115.	ALU 247/115 was higher in patients who achieved a pathological complete response and correlated with a better DFS.	Predictive (Response NACT) Prognostic (DFS)	Giro et al., 2024 [52]
10 BC 26 BBD 22 HC	BC = 45 BBD = 44 HC = 44	Plasma	qPCR on ALU 247 bp and ALU 115 bp. cfDI calculated as the ratio of ALU 247/115.	ALU 247/115 was higher in high-grade and ER+ tumours.	Prognostic (Tumour grade and ER+)	Gameel et al., 2024 [53]

**Table 2:** Summary of methods and main results of articles on the integrity of circulating cell-free DNA in prostate cancer. Abbreviations: BDUT: benign disease of urogenital tract; BPH: benign prostate hyperplasia; cfDI: circulating cell-free DNA integrity; HC: healthy control; MPCa: metastatic prostate cancer; nMPCa: non-metastatic prostate cancer; PCa: prostate cancer.

Number of Patients or Controls	Average Age (yr) Patients or Controls	Analysed Samples	Methods and Targets	Main Results	Clinical Value of cfDI	Citations
61 PCa 62 BPH	PCa = 69 BPH = 66	Plasma	qPCR on LEP 356 bp and LEP 105 bp. cfDI calculated as the ratio of LEP 356/105.	There was no difference in LEP 356/105 between PCa and BPH.	No	Boddy et al., 2006 [55]
123 PCa 20 HC under 40 yrs (Ctr1) 25 patients with radical prostatectomy (Ctr2) 22 patients with negative prostate biopsy (Ctr3)	PCa = 59 Ctr1 = 32 Ctr2 = 58 Ctr3 = 61	Plasma	qPCR on 1.3, 1.8, and 2.4 kb fragments from four genomic loci (12 conditions). cfDI calculated as the sum of the individual results, expressed as 0 (qPCR negativity) or 1 (qPCR positivity).	The cfDI of the PCa group was higher than that of the Ctr1 and Ctr2 groups but not higher than that of the Ctr3 group.	Diagnostic (PCa vs. HC or after Radical Prostatectomy)	Hanley et al., 2006 [56]
96 PCa 112 BPH	PCa = 63 BPH = 60	Plasma	qPCR on ALU 247 bp and ALU 115 bp. cfDI calculated as the ratio of ALU 247/ALU 115.	ALU 247/115 was higher in PCa than in BPH. This difference was retained also in PCa and BPH with PSA > 4 ng/mL.	Diagnostic (PCa vs. BPH)	Feng et al., 2013 [57]
29 PCa 25 HC	PCa = 65 HC = 66	Urine	qPCR on fragments longer than 250 bp from c-MYC, BCAS1, and HER2 genes. cfDI calculated as the sum of the three genes quantification.	The cfDI was higher in PCa than in HC.	Diagnostic (PCa vs. HC)	Casadio et al., 2013 [58]
67 PCa 64 BDUT	PCa = 68 BDUT = 62	Urine	qPCR on fragments longer than 250 bp of the genes c-MYC, AR, and HER2. cfDI calculated as the sum of the quantification of the three genes.	There was no difference in cfDI between PCa and BDUT.	No	Salvi et al., 2015 [59]
50 PCa (28 MPCa, 22 nMPCa) 25 BPH 30 HC	PCa = 66 BPH = 69 HC = 62	Plasma	qPCR on ALU 247 bp and ALU 115 bp. cfDI calculated as the ratio of ALU 247/115.	ALU 247/115 was higher in PCa than in BPH or HC. The cfDI was slightly higher in nMPCa than in MPCa.	Diagnostic (PCa vs. BPH or HC) and Prognostic (Metastatic Disease)	Fawzy et al., 2016 [60]
30 PCa (10 MPCa, 20 nMPCa), 40 BPH 30 HC	PCa = 64 BPH = 61 HC = 61	Plasma	qPCR on ALU 247 bp and ALU 115 bp. cfDI calculated as the ratio of ALU 247/115.	ALU 247/115 was higher in PCa than in BPH or HC. There was no difference in cfDI between MPCa and nMPCa.	Diagnostic (PCa vs. BPH or HC)	Khani et al., 2019 [61]
31 PCa 30 HC	PCa = 71 HC = 56	Serum	qPCR on ALU 247 bp and ALU 115 bp. cfDI calculated as the ratio of ALU 247/115.	ALU 247/115 was higher in PCa than in HC. The cfDI increases with tumour stage.	Diagnostic (PCa vs. HC) and Prognostic (Tumours Stage)	Arko-Boham et al., 2019 [41]
11 PCa 9 BPH	PCa = 68 BPH = 65	Plasma	qPCR on ALU 247 bp and ALU 115 bp. cfDI calculated as the ratio of ALU 247/115.	There was no difference in ALU 247/115 between PCa and BPH.	No	Condappa et al., 2020 [62]

## References

[1] Guo L., Kong D., Liu J., Zhan L., Luo L., Zheng W., Zheng Q., Chen C., Sun S. (2023). Breast Cancer Heterogeneity and Its Implication in Personalized Precision Therapy. Exp. Hematol. Oncol..

[2] Bray F., Laversanne M., Sung H., Ferlay J., Siegel R.L., Soerjomataram I., Jemal A. (2024). Global Cancer Statistics 2022: GLOBOCAN Estimates of Incidence and Mortality Worldwide for 36 Cancers in 185 Countries. CA Cancer J. Clin..

[3] Saadatmand S., Bretveld R., Siesling S., Tilanus-Linthorst M.M.A. (2015). Influence of Tumour Stage at Breast Cancer Detection on Survival in Modern Times: Population Based Study in 173,797 Patients. BMJ.

[4] Ginsburg O., Yip C.H., Brooks A., Cabanes A., Caleffi M., Yataco J.A.D., Gyawali B., McCormack V., de Anderson M.M.L., Mehrotra R. (2020). Breast Cancer Early Detection: A Phased Approach to Implementation. Cancer.

[5] Thill M., Kolberg-Liedtke C., Albert U.S., Banys-Paluchowski M., Bauerfeind I., Blohmer J.U., Budach W., Dall P., Ditsch N., Fallenberg E.M. (2023). AGO Recommendations for the Diagnosis and Treatment of Patients with Locally Advanced and Metastatic Breast Cancer: Update 2023. Breast Care.

[6] Speirs V. (2021). Quality Considerations When Using Tissue Samples for Biomarker Studies in Cancer Research. Biomark. Insights.

[7] Armakolas A., Kotsari M., Koskinas J. (2023). Liquid Biopsies, Novel Approaches and Future Directions. Cancers.

[8] Alimirzaie S., Bagherzadeh M., Akbari M.R. (2019). Liquid Biopsy in Breast Cancer: A Comprehensive Review. Clin. Genet..

[9] Lüönd F., Tiede S., Christofori G. (2021). Breast Cancer as an Example of Tumour Heterogeneity and Tumour Cell Plasticity during Malignant Progression. Br. J. Cancer.

[10] Zhang W., Cao G., Wu F., Wang Y., Liu Z., Hu H., Xu K. (2023). Global Burden of Prostate Cancer and Association with Socioeconomic Status, 1990–2019: A Systematic Analysis from the Global Burden of Disease Study. J. Epidemiol. Glob. Health.

[11] Schwarzenbach H., Hoon D.S.B., Pantel K. (2011). Cell-Free Nucleic Acids as Biomarkers in Cancer Patients. Nat. Rev. Cancer.

[12] Ionescu F., Zhang J., Wang L. (2022). Clinical Applications of Liquid Biopsy in Prostate Cancer: From Screening to Predictive Biomarker. Cancers.

[13] Mazzitelli C., Santini D., Corradini A.G., Zamagni C., Trerè D., Montanaro L., Taffurelli M. (2023). Liquid Biopsy in the Management of Breast Cancer Patients: Where Are We Now and Where Are We Going. Diagnostics.

[14] Alix-Panabières C., Pantel K. (2021). Liquid Biopsy: From Discovery to Clinical Application. Cancer Discov..

[15] Sánchez-Herrero E., Serna-Blasco R., Robado de Lope L., González-Rumayor V., Romero A., Provencio M. (2022). Circulating Tumor DNA as a Cancer Biomarker: An Overview of Biological Features and Factors That May Impact on CtDNA Analysis. Front. Oncol..

[16] Huang J., Huang D., Ruan X., Zhan Y., Chun S.T.-T., Ng A.T.-L., Na R. (2023). Clinical Translational Research of Liquid Biopsy Applications in Prostate Cancer and Other Urological Cancers. Camb. Prism. Precis. Med..

[17] Ma F., Guan Y., Yi Z., Chang L., Li Q., Chen S., Zhu W., Guan X., Li C., Qian H. (2020). Assessing Tumor Heterogeneity Using CtDNA to Predict and Monitor Therapeutic Response in Metastatic Breast Cancer. Int. J. Cancer.

[18] Freitas A.J.A.d., Causin R.L., Varuzza M.B., Calfa S., Hidalgo Filho C.M.T., Komoto T.T., Souza C.d.P., Marques M.M.C. (2022). Liquid Biopsy as a Tool for the Diagnosis, Treatment, and Monitoring of Breast Cancer. Int. J. Mol. Sci.

[19] Urabe F., Sumiyoshi T., Tashiro K., Goto T., Kimura T., Kobayashi T. (2024). Prostate Cancer and Liquid Biopsies: Clinical Applications and Challenges. Int. J. Urol..

[20] Obinata D., Yamada Y., Sumiyoshi T., Tanegashima T., Watanabe R., Kobayashi H., Ito D., Urabe F., Japanese Young Urologist Basic Research Collaboration (2024). Recent Advances in Basic Research on Prostate Cancer: Where We Are Heading?. Int. J. Urol..

[21] Marvalim C., Datta A., Lee S.C. (2023). Role of P53 in Breast Cancer Progression: An Insight into P53 Targeted Therapy. Theranostics.

[22] Mollon L.E., Anderson E.J., Dean J.L., Warholak T.L., Aizer A., Platt E.A., Tang D.H., Davis L.E. (2020). A Systematic Literature Review of the Prognostic and Predictive Value of PIK_3_CA Mutations in HR^+^/HER2^−^ Metastatic Breast Cancer. Clin. Breast Cancer.

[23] Agostini M., Enzo M.V., Bedin C., Belardinelli V., Goldin E., Del Bianco P., Maschietto E., D’Angelo E., Izzi L., Saccani A. (2012). Circulating Cell-Free DNA: A Promising Marker of Regional Lymphonode Metastasis in Breast Cancer Patients. Cancer Biomark..

[24] Stötzer O.J., Lehner J., Fersching-Gierlich D., Nagel D., Holdenrieder S. (2014). Diagnostic Relevance of Plasma DNA and DNA Integrity for Breast Cancer. Tumour Biol..

[25] Cristiano S., Leal A., Phallen J., Fiksel J., Adleff V., Bruhm D.C., Jensen S.Ø., Medina J.E., Hruban C., White J.R. (2019). Genome-Wide Cell-Free DNA Fragmentation in Patients with Cancer. Nature.

[26] Umetani N., Giuliano A.E., Hiramatsu S.H., Amersi F., Nakagawa T., Martino S., Hoon D.S.B. (2006). Prediction of Breast Tumor Progression by Integrity of Free Circulating DNA in Serum. J. Clin. Oncol..

[27] Deligezer U., Eralp Y., Akisik E.Z., Akisik E.E., Saip P., Topuz E., Dalay N. (2008). Effect of Adjuvant Chemotherapy on Integrity of Free Serum DNA in Patients with Breast Cancer. Ann. N. Y. Acad. Sci..

[28] Deligezer U., Eralp Y., Akisik E.E., Akisik E.Z., Saip P., Topuz E., Dalay N. (2008). Size Distribution of Circulating Cell-Free DNA in Sera of Breast Cancer Patients in the Course of Adjuvant Chemotherapy. Clin. Chem. Lab. Med..

[29] Lehner J., Stötzer O.J., Fersching D., Nagel D., Holdenrieder S. (2013). Circulating Plasma DNA and DNA Integrity in Breast Cancer Patients Undergoing Neoadjuvant Chemotherapy. Clin. Chim. Acta.

[30] Madhavan D., Wallwiener M., Bents K., Zucknick M., Nees J., Schott S., Cuk K., Riethdorf S., Trumpp A., Pantel K. (2014). Plasma DNA Integrity as a Biomarker for Primary and Metastatic Breast Cancer and Potential Marker for Early Diagnosis. Breast Cancer Res. Treat..

[31] Iqbal S., Vishnubhatla S., Raina V., Sharma S., Gogia A., Deo S.S.V., Mathur S., Shukla N.K. (2015). Circulating Cell-Free DNA and Its Integrity as a Prognostic Marker for Breast Cancer. SpringerPlus.

[32] Kamel A.M., Teama S., Fawzy A., El Deftar M. (2016). Plasma DNA Integrity Index as a Potential Molecular Diagnostic Marker for Breast Cancer. Tumour Biol..

[33] Maltoni R., Casadio V., Ravaioli S., Foca F., Tumedei M.M., Salvi S., Martignano F., Calistri D., Rocca A., Schirone A. (2017). Cell-Free DNA Detected by “Liquid Biopsy” as a Potential Prognostic Biomarker in Early Breast Cancer. Oncotarget.

[34] Wang W., Liang M., Ma G., Li L., Zhou W., Xia T., Xie H., Wang S. (2017). Plasma Cell-Free DNA Integrity plus Circulating Tumor Cells: A Potential Biomarker of No Distant Metastasis Breast Cancer. Neoplasma.

[35] Cheng J., Cuk K., Heil J., Golatta M., Schott S., Sohn C., Schneeweiss A., Burwinkel B., Surowy H. (2017). Cell-Free Circulating DNA Integrity Is an Independent Predictor of Impending Breast Cancer Recurrence. Oncotarget.

[36] Cheng J., Holland-Letz T., Wallwiener M., Surowy H., Cuk K., Schott S., Trumpp A., Pantel K., Sohn C., Schneeweiss A. (2018). Circulating Free DNA Integrity and Concentration as Independent Prognostic Markers in Metastatic Breast Cancer. Breast Cancer Res. Treat..

[37] Tang Z., Li L., Shen L., Shen X., Ju S., Cong H. (2018). Diagnostic Value of Serum Concentration and Integrity of Circulating Cell-Free DNA in Breast Cancer: A Comparative Study With CEA and CA15-3. Lab. Med..

[38] Salimi M., Burkhani S.S. (2019). Integrity and Quantity Evaluation of Plasma Cell-Free DNA in Triple Negative Breast Cancer. Avicenna J. Med. Biotechnol..

[39] Wang W., Zhang W., Su L., Sang J., Wang S., Yao Y. (2019). Plasma Cell-Free DNA Integrity: A Potential Biomarker to Monitor the Response of Breast Cancer to Neoadjuvant Chemotherapy. Transl. Cancer Res..

[40] Miao Y., Fan Y., Zhang L., Ma T., Li R. (2019). Clinical Value of Plasma CfDNA Concentration and Integrity in Breast Cancer Patients. Cell. Mol. Biol..

[41] Arko-Boham B., Aryee N.A., Blay R.M., Owusu E.D.A., Tagoe E.A., Doris Shackie E.S., Debrah A.B., Adu-Aryee N.A. (2019). Circulating Cell-Free DNA Integrity as a Diagnostic and Prognostic Marker for Breast and Prostate Cancers. Cancer Genet..

[42] Hussein N.A., Mohamed S.N., Ahmed M.A. (2019). Plasma ALU-247, ALU-115, and CfDNA Integrity as Diagnostic and Prognostic Biomarkers for Breast Cancer. Appl. Biochem. Biotechnol..

[43] Park M.K., Lee J.C., Lee J.W., Hwang S.J. (2021). Alu Cell-Free DNA Concentration, Alu Index, and LINE-1 Hypomethylation as a Cancer Predictor. Clin. Biochem..

[44] Lamminaho M., Kujala J., Peltonen H., Tengström M., Kosma V.M., Mannermaa A. (2021). High Cell-Free DNA Integrity Is Associated with Poor Breast Cancer Survival. Cancers.

[45] Adusei E., Ahenkorah J., Adu-Aryee N.A., Adutwum-Ofosu K.K., Tagoe E.A., Koney N.K.K., Nkansah E., Aryee N.A., Blay R.M., Hottor B.A. (2021). Reduced Serum Circulation of Cell-Free DNA Following Chemotherapy in Breast Cancer Patients. Med. Sci..

[46] Cirmena G., Ferrando L., Ravera F., Garuti A., Dameri M., Gallo M., Barbero V., Ferrando F., Del Mastro L., Garlaschi A. (2022). Plasma Cell-Free DNA Integrity Assessed by Automated Electrophoresis Predicts the Achievement of Pathologic Complete Response to Neoadjuvant Chemotherapy in Patients with Breast Cancer. JCO Precis. Oncol..

[47] Elhelaly R., Effat N., Hegazy M.A.E.F., Abdelwahab K., Hamdy O., Hashem E.M.A., Elzehery R.R. (2022). Circulating Cell Free DNA and DNA Integrity Index as Discriminating Tools between Breast Cancer and Benign Breast Disease. Asian Pac. J. Cancer Prev..

[48] Abd El Hafeez H.A., Abd El Rahman M.Z., Kamel T.M., Rezk K.M., Mohamed F.M., Abdel-Hameed Z.A. (2023). The Role of Circulating Cell-Free DNA and Its Integrity as a Biomarker for Diagnosis of Breast Cancer Using ALU (247/115) BP Sequences. Egypt. J. Immunol..

[49] Nair M.G., Ramesh R.S., Naidu C.M., Mavatkar A.D., Snijesh V.P., Ramamurthy V., Somashekaraiah V.M., Anupama C.E., Raghunathan K., Panigrahi A. (2023). Estimation of ALU Repetitive Elements in Plasma as a Cost-Effective Liquid Biopsy Tool for Disease Prognosis in Breast Cancer. Cancers.

[50] Bortul M., Giudici F., Tierno D., Generali D., Scomersi S., Grassi G., Bottin C., Cappelletti M.R., Zanconati F., Scaggiante B. (2023). A Case–Control Study by DdPCR of ALU 260/111 and LINE-1 266/97 Copy Number Ratio in Circulating Cell-Free DNA in Plasma Revealed LINE-1 266/97 as a Potential Biomarker for Early Breast Cancer Detection. Int. J. Mol. Sci..

[51] Çelik B., Peker Eyüboğlu İ., Koca S., Ümit Uğurlu M., Alan Ö., Güllü Amuran G., Akin Telli T., Yumuk F., Akkiprik M. (2024). Correlation between Plasma CcfDNA, MtDNA Changes, CTCs, and Epithelial-Mesenchymal Transition in Breast Cancer Patients Undergoing NACT. Turk. J. Med. Sci..

[52] Giro C., Yamada A.M.T.D., Cruz F.J.S.M., Lilian L.A., Alves B.d.C.A., Fonseca F.L.A., del Giglio A. (2024). Measuring CfDNA Integrity as a Biomarker for Predicting Neoadjuvant Chemotherapy Response in Breast Cancer Patients: A Pilot Study. Breast Cancer Res. Treat..

[53] Gameel A.M., Talaat R.M., Sakr M.A., Selim M.A., Abo Alil D.F.A., Elkhouly E.A. (2024). Circulating Tumor DNA in Egyptian Women with Breast Cancer: A Marker for Detection of Primary Cases and Early Prediction of Recurrence. Clin. Chim. Acta.

[54] Kanagaraju V., Ashlyin P.V.K., Elango N., Devanand B. (2020). Role of Transrectal Ultrasound Elastography in the Diagnosis of Prostate Carcinoma. J. Med. Ultrasound.

[55] Boddy J.L., Gal S., Malone P.R., Shaida N., Wainscoat J.S., Harris A.L. (2006). The Role of Cell-Free DNA Size Distribution in the Management of Prostate Cancer. Oncol. Res..

[56] Hanley R., Rieger-Christ K.M., Canes D., Emara N.R., Shuber A.P., Boynton K.A., Libertino J.A., Summerhayes I.C. (2006). DNA Integrity Assay: A Plasma-Based Screening Tool for the Detection of Prostate Cancer. Clin. Cancer Res..

[57] Feng J., Feng G., Xiao L., Tang J., Huang H., Cao Y., Li G. (2013). Plasma Cell-Free DNA and Its DNA Integrity as Biomarker to Distinguish Prostate Cancer from Benign Prostatic Hyperplasia in Patients with Increased Serum Prostate-Specific Antigen. Int. Urol. Nephrol..

[58] Casadio V., Calistri D., Salvi S., Gunelli R., Carretta E., Amadori D., Silvestrini R., Zoli W. (2013). Urine Cell-Free DNA Integrity as a Marker for Early Prostate Cancer Diagnosis: A Pilot Study. Biomed. Res. Int..

[59] Salvi S., Gurioli G., Martignano F., Foca F., Gunelli R., Cicchetti G., De Giorgi U., Zoli W., Calistri D., Casadio V. (2015). Urine Cell-Free DNA Integrity Analysis for Early Detection of Prostate Cancer Patients. Dis. Markers.

[60] Fawzy A., Sweify K.M., El-Fayoumy H.M., Nofal N. (2016). Quantitative Analysis of Plasma Cell-Free DNA and Its DNA Integrity in Patients with Metastatic Prostate Cancer Using ALU Sequence. J. Egypt. Natl. Cancer Inst..

[61] Khani M., Hosseini J., Mirfakhraie R., Habibi M., Azargashb E., Pouresmaeili F. (2019). The Value of the Plasma Circulating Cell-Free DNA Concentration and Integrity Index as a Clinical Tool for Prostate Cancer Diagnosis: A Prospective Case-Control Cohort Study in an Iranian Population. Cancer Manag. Res..

[62] Condappa A., McGrowder D., Aiken W., McLaughlin W., Gossell-Williams M. (2020). Evaluation of Plasma Circulating Cell Free DNA Concentration and Integrity in Patients with Prostate Cancer in Jamaica: A Preliminary Study. Diseases.

[63] Caruso A., Gelsomino L., Panza S., Accattatis F.M., Naimo G.D., Barone I., Giordano C., Catalano S., Andò S. (2023). Leptin: A Heavyweight Player in Obesity-Related Cancers. Biomolecules.

[64] De Mattos-Arruda L., Caldas C. (2016). Cell-Free Circulating Tumour DNA as a Liquid Biopsy in Breast Cancer. Mol. Oncol..

[65] Műzes G., Bohusné Barta B., Szabó O., Horgas V., Sipos F. (2022). Cell-Free DNA in the Pathogenesis and Therapy of Non-Infectious Inflammations and Tumors. Biomedicines.

[66] Madsen A.T., Hojbjerg J.A., Sorensen B.S., Winther-Larsen A. (2019). Day-to-Day and within-Day Biological Variation of Cell-Free DNA. EBioMedicine.

[67] Gezer U., Bronkhorst A.J., Holdenrieder S. (2022). The Utility of Repetitive Cell-Free DNA in Cancer Liquid Biopsies. Diagnostics.

[68] Serra R., Giunta E.F., Schepisi G., Brighi N., Montanari D., Lolli C., Bleve S., Piras M., Palmieri G., Scartozzi M. (2024). An Evaluation of Talazoparib plus Enzalutamide for the Treatment of Metastatic Castration-Resistant Prostate Cancer. Expert Rev. Anticancer Ther..

[69] Kostos L., Tran B., Azad A.A. (2024). Combination of PARP Inhibitors and Androgen Receptor Pathway Inhibitors in Metastatic Castration-Resistant Prostate Cancer. Drugs.

[70] Yang J., Qiu L., Wang X., Chen X., Cao P., Yang Z., Wen Q. (2023). Liquid Biopsy Biomarkers to Guide Immunotherapy in Breast Cancer. Front. Immunol..

[71] Kitahara M., Hazama S., Tsunedomi R., Takenouchi H., Kanekiyo S., Inoue Y., Nakajima M., Tomochika S., Tokuhisa Y., Iida M. (2016). Prediction of the Efficacy of Immunotherapy by Measuring the Integrity of Cell-Free DNA in Plasma in Colorectal Cancer. Cancer Sci..

[72] Waki K., Yokomizo K., Yoshiyama K., Takamori S., Komatsu N., Yamada A. (2021). Integrity of Circulating Cell-Free DNA as a Prognostic Biomarker for Vaccine Therapy in Patients with Nonsmall Cell Lung Cancer. Immunopharmacol. Immunotoxicol..

